# *Streptococcus pneumoniae* interactions with the complement system

**DOI:** 10.3389/fcimb.2022.929483

**Published:** 2022-07-28

**Authors:** Eliza Gil, Mahdad Noursadeghi, Jeremy S. Brown

**Affiliations:** ^1^ Division of Infection and Immunity, University College London, London, United Kingdom; ^2^ Division of Medicine, University College London, London, United Kingdom

**Keywords:** *Streptococcus pneumoniae*, streptococcal infections, complement - immunological terms, immune evasion, innate immunity

## Abstract

Host innate and adaptive immunity to infection with *Streptococcus pneumoniae* is critically dependent on the complement system, demonstrated by the high incidence of invasive *S. pneumoniae* infection in people with inherited deficiency of complement components. The complement system is activated by *S. pneumoniae* through multiple mechanisms. The classical complement pathway is activated by recognition of *S. pneumoniae* by C-reactive protein, serum amyloid P, C1q, SIGN-R1, or natural or acquired antibody. Some *S. pneumoniae* strains are also recognised by ficolins to activate the mannose binding lectin (MBL) activation pathway. Complement activation is then amplified by the alternative complement pathway, which can also be activated by *S. pneumoniae* directly. Complement activation results in covalent linkage of the opsonic complement factors C3b and iC3b to the *S. pneumoniae* surface which promote phagocytic clearance, along with complement-mediated immune adherence to erythrocytes, thereby protecting against septicaemia. The role of complement for mucosal immunity to *S. pneumoniae* is less clear. Given the major role of complement in controlling infection with *S. pneumoniae*, it is perhaps unsurprising that *S. pneumoniae* has evolved multiple mechanisms of complement evasion, including the capsule, multiple surface proteins, and the toxin pneumolysin. There is considerable variation between *S. pneumoniae* capsular serotypes and genotypes with regards to sensitivity to complement which correlates with ability to cause invasive infections. However, at present we only have a limited understanding of the main mechanisms causing variations in complement sensitivity between *S. pneumoniae* strains and to non-pathogenic streptococci.

## Introduction

*Streptococcus pneumoniae* colonises the nasopharynx of a high proportion of infants and up to 10% of adults. *S. pneumoniae* can spread from the nasopharynx to the lungs to cause pneumonia, the blood to cause septicaemia (often associated with pneumonia), or to the meninges to cause meningitis and is a common cause of severe infections worldwide (Troeger et al., 2018). Similar to other mainly extracellular bacterial pathogens, host immunity to *S. pneumoniae* is critically dependent on phagocytosis and therefore opsonisation by circulating soluble host immune mediators, particularly complement. The importance of complement for prevention of *S. pneumoniae* infection is demonstrated by the high incidence of invasive *S. pneumoniae* infections people with complement deficiencies (Sjöholm et al., 2006; Skattum et al., 2011; Woehrl et al., 2011). For example, around 50% of subjects with inherited deficiency of the classical complement pathway component C2 have had an episode of pneumonia, septicaemia, or meningitis, the majority of which were caused by *S. pneumoniae* (Jönsson et al., 2005). Genome wide association studies showing an association between genotypic complement variants and poor outcomes in *S. pneumoniae* meningitis provide additional evidence of the importance of complement for immunity to *S. pneumoniae* (Weiss et al., 1998; Biesma et al., 2001). Furthermore, human infections with *S. pneumoniae* are associated with significant reductions in circulating levels of complement factors (Coonrod and Rylko-Bauer, 1977) suggesting complement activation and consumption.

## Complement activation by *S. pneumoniae*

**Figure 1:**
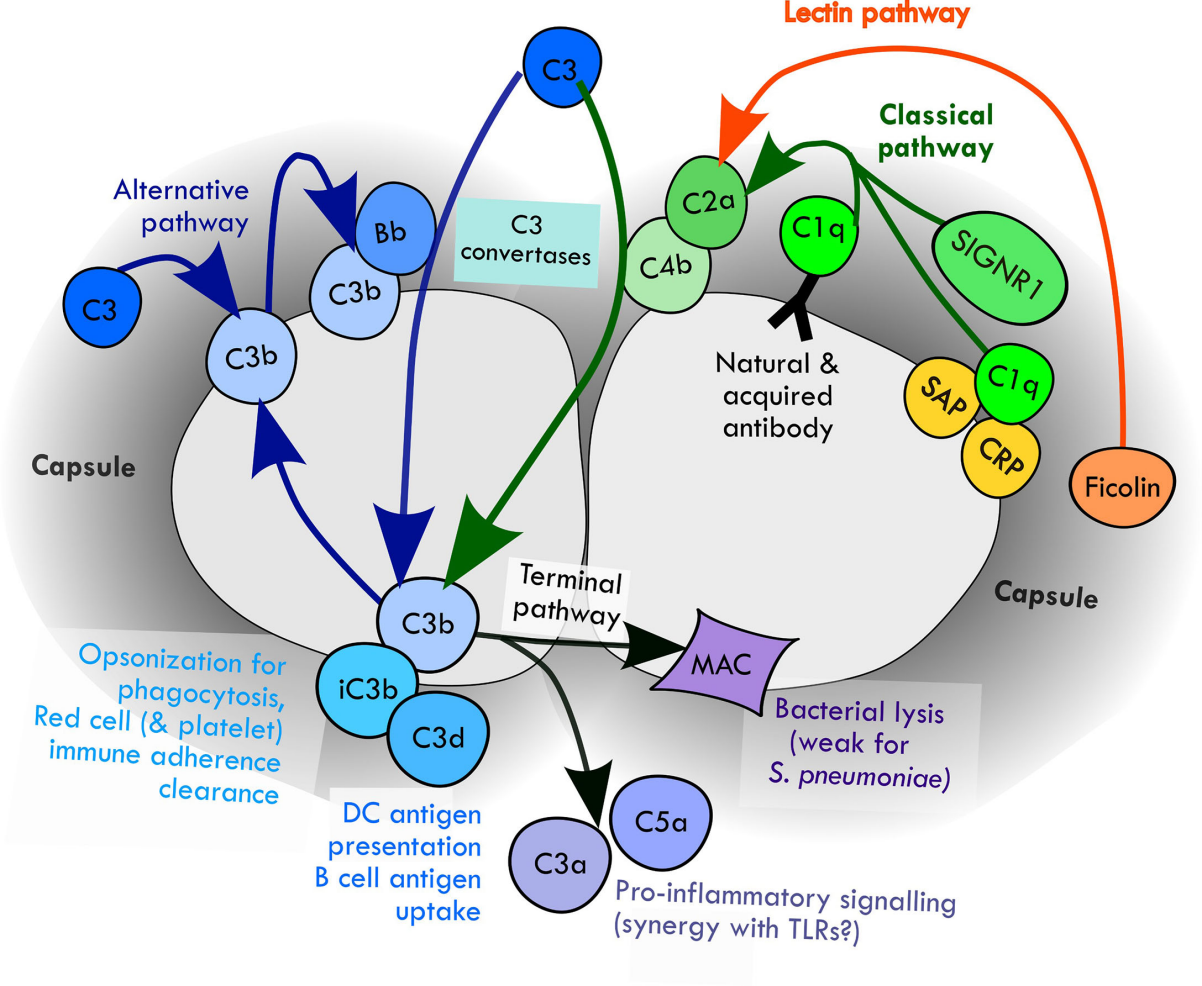
Mechanisms of activation of complement pathways by *S. pneumoniae* and the downstream potential effects on the immune response.

The complement system is activated by three mechanisms: the classical, alternative and lectin pathways (Walport, 2001). Data obtained using genetically modified mice or serum from human subjects with complement deficiencies have demonstrated a key role for the classical pathway for initiation of complement deposition on *S. pneumoniae*. Classical pathway deficiency reduced opsonisation with complement and complement-mediated phagocytosis of *S. pneumoniae* by neutrophils, and increased target organ CFU and disease severity in mouse models of pneumonia, septicaemia and meningitis (Brown et al., 2002; Rupprecht et al., 2007; Yuste et al., 2008). Although the classical pathway was previously considered to be activated by antibody and therefore mediate adaptive immunity, mouse data have demonstrated an essential role for the classical pathway for innate complement-mediated immunity to *S. pneumoniae* (Brown et al., 2002). Further work has demonstrated that multiple pathways activate classical pathway recognition of *S. pneumoniae* independent of acquired antibody, including direct binding of C1q (the first complement of the classical pathway), natural antibody recognition (mainly of cell wall phosphocholine), binding of the serum components C-reactive protein (CRP) and serum amyloid P (SAP) protein, and activation on splenic macrophage cell surfaces through SIGN-R1 recognition of the *S. pneumoniae* capsule (**Figure 1**) (Brown et al., 2002; Kang et al., 2004; Rupprecht et al., 2007; Suresh et al., 2007; Yuste et al., 2007; Agrawal et al., 2008)

The alternative pathway amplifies complement activation by *S. pneumoniae* initiated by the classical and lectin pathways (Xu et al., 2001; Brown et al., 2002) and is also activated directly through recognition of cell wall phosphocholine (Winkelstein and Tomasz, 1978; Hummell et al., 1981; Brown et al., 1983b; Hummell et al., 1985; Hyams et al., 2010). Mice with deficiencies in components of the alternative pathway show impaired opsonophagocytosis of *S. pneumoniae* and increased susceptibility to infection (Xu et al., 2001; Brown et al., 2002; Li et al., 2011). In humans, hereditary deficiency of the alternative pathway component factor D is associated with severe pneumococcal disease (Weiss et al., 1998; Biesma et al., 2001). The role of the lectin complement pathway for protection against *S. pneumoniae* infection is less clear. The lectin pathway is activated by the binding to microbial carbohydrates of mannose-binding lectin (MBL) and the ficolins (Krarup et al., 2005). Meta-analysis has suggested MBL deficiency in humans is associated with an increased susceptibility to invasive *S. pneumoniae* infections (Eisen et al., 2008; Brouwer et al., 2009) and L-ficolin binds to at least some capsular serotypes of *S. pneumoniae* (Krarup et al., 2005). However, data from genetically modified mice is contradictory with some data showing the lectin pathway only has a small role in complement recognition of *S. pneumoniae* (Neth et al., 2000; Brown et al., 2002; Krarup et al., 2005; Brady et al., 2014) but other data demonstrating deficiency of MASP-2, the effector enzyme of the lectin pathway, increases susceptible to *S. pneumoniae* (Ali et al., 2012).

## Complement mediated immunity to *S. pneumoniae*

Recognition of *S. pneumoniae* by the classical, alternative and lectin pathways results in activation of protease cascades that converge on the central complement component C3 (**Figure 1**). Cleavage of C3 by C3 convertases results in formation of the opsonins C3b and iC3b, along with activation of the terminal complement pathway. This results in the three main mechanisms by which complement directly prevents infection: (1) opsonisation and increased phagocytosis of the pathogen by covalent linkage of C3b and iC3b to bacterial surface molecules; (2) pro-inflammatory signaling by the complement components C3a and C5a; and (3) direct killing of the pathogen by formation of the membrane attack complex, a lipophilic complex of the terminal complement components which forms a pore in the pathogen phospholipid bilayer leading to bacterial lysis (Walport, 2001). Opsonisation with C3b/iC3b strongly promotes both innate and adaptive (mediated by acquired antibody) clearance of *S. pneumoniae* by phagocytosis (Gordon et al., 1986; van Lookeren Campagne et al., 2007; Hyams et al., 2010; Hyams et al., 2013; Wilson et al., 2017). C5a production in response to *S. pneumoniae* increases cytokine production by human white cells, possibly involving cross-talk with toll like receptor-mediated inflammatory responses (van der Maten et al., 2016). Some data also suggest complement-mediated inflammation is in fact detrimental in meningitis through promoting host neurotoxicity (Kasanmoentalib et al., 2017). *S. pneumoniae* is generally resistant to MAC-mediated killing (Joiner et al., 1983).

Along with direct effects on innate immunity, additional mechanisms by which complement promotes immunity to bacteria have been described. Red cells express complement receptor 1 (CR1), and thereby bind *S. pneumoniae* opsonised with C3b and transport them to the liver and spleen to be phagocytosed by tissue macrophages (Li et al., 2009; van Lookeren Campagne and Verschoor, 2018). In transgenic mice expressing human CR1 on red cells this mechanism promotes clearance of *S. pneumoniae* from the blood (Li et al., 2009). A similar mechanism of bacterial clearance involving bacterial adhesion to platelets *via* CR1 has also been described for other pathogens (Broadley et al., 2016), but it is not known whether this is relevant for *S. pneumoniae*. Recent data has also shown that complement-opsonised *S. pneumoniae* captured by splenic macrophages can then be killed by splenic neutrophils (Deniset et al., 2017). In addition, opsonisation with C3b also promotes adaptive antibody responses to *S. pneumoniae* by improving antigen presentation by dendritic cells and antigen uptake by B cells (Griffioen et al., 1991; Peset Llopis et al., 1996; Haas et al., 2002; Donius et al., 2013; Heesters and Carroll, 2016). Other complement dependent mechanisms of immunity that have recently been described include intracellular activation of pro-inflammatory pathways, bacterial killing *via* autophagy, and improved activation of T cells (Reis et al., 2019), but there are only limited or no clear data for their relevance for *S. pneumoniae.*

There are also likely to be differences in the role or significance of complement-mediated immunity to *S. pneumoniae* in different anatomical sites. The high incidence of septicaemia, meningitis, pneumoniae and otitis media in humans with inherited complement deficiencies (Skattum et al., 2011) suggest complement is required for prevention of both systemic and respiratory tract infections. Mouse infection models data convincingly show complement has a very powerful role in preventing septicaemia, with a less important but significant role for protecting against lung infection and otitis media (Brown et al., 2002; Ren et al., 2004b; Yuste et al., 2005; Bogaert et al., 2009; Sabharwal et al., 2009). Experiments using complement deficient mice suggest complement has a limited role in promoting clearance of *S. pneumoniae* in simple models of nasopharyngeal colonisation (van Rossum et al., 2005; Bogaert et al., 2010). However, in a dual infection model *Haemophilus influenzae* stimulated *S. pneumoniae* clearance from the nasopharynx and this required complement, suggesting that complement may help limit *S. pneumoniae* colonisation when the nasopharynx is inflamed (Lysenko et al., 2005). How important complement-mediated inflammation is for the pathogenesis *S. pneumoniae* infections at different anatomical sites is not clear and requires further investigation.

## Mechanisms of complement evasion by *S. pneumoniae*

The importance of complement for immunity to *S. pneumoniae* is also demonstrated by the multiple mechanisms *S. pneumoniae* has evolved to evade complement mediated immunity, several of which are important for *S. pneumoniae* to establish invasive infection. The key mechanisms used by *S. pneumoniae* to inhibit complement-mediated immunity are described below and summarised in **Table 1**.

**Table 1:** Mechanisms of inhibition of complement activity by *S. pneumoniae*.

S. pneumoniae factor	Mechanism	Pathway(s) affected*	Reference
Capsule	Reduces surface binding of: *Antibody: natural and adaptive *CRP *Complement factors Inhibits conversion of C3b to iC3b Impairs C3b/iC3b binding to complement receptors Chemical structure not recognized by ficolins	CP CP AP Opsonisation Opsonisation LP	Brown et al., 1983a Joiner et al., 1983 Hyams et al., 2010 Brady et al., 2014
Physical factors	Growth as diplococci rather than chains limits bacteria opsonized by limited sites of complement activation	CP and AP	Dalia and Weiser, 2011 Pathak et al., 2018
Pneumolysin	Binds C1q in serum to divert complement activity	CP	Paton et al., 1984 Mitchell et al., 1991 Yuste et al., 2005
PspA	Inhibits CRP deposition on bacterial surface Impairs C3 convertases formation Sequesters complement regulatory protein C4BP	CP CP and AP CP	Jedrzejas, 2001 Ren et al., 2004a Yuste et al., 2005 Mukerji et al., 2012 Haleem et al., 2019
PspC	Binds factor H May bind C4BP	AP CP	Dave et al., 2004 Quin et al., 2005 Hammerschmidt et al., 2007 Li et al., 2007 Yuste et al., 2010 Haleem et al., 2019
LytA and C	Binds factor H Binds C4BP Inhibit C1q binding to CRP Cleave C3b and iC3b bound to bacterial surface	AP CP CP Opsonisation	Ramos-Sevillano et al., 2011 Ramos-Sevillano et al., 2015
Phts	Binds factor H Direct degradation of C3	AP CP and AP	Zhang et al., 2001 Ogunniyi et al., 2009
BgaA, Nan and StrH	Deglycosylation of complement protein glycoconjugates	CP and AP	Dalia et al., 2010
PepO	Indirectly degrades C3b Binds and depletes C1q	Opsonisation CP	Agarwal et al., 2013 Agarwal et al., 2014
Tuf	Binds factor H	AP	Mohan et al., 2014
GAPDH	Binds and depletes C1q	CP	Terrasse et al., 2012
PGK	Inhibits MAC formation	MAC	Blom et al., 2014
Eno	Binds C4BP	CP	Agarwal et al., 2012

*CP, classical pathway; LP, lectin pathway; AP, alternative pathway; MAC, inhibition of MAC.

### The capsule

Virulent strains of *S. pneumoniae* capable of causing invasive infections have an external capsular layer made of repeating chains of polysaccharide anchored to the peptidoglycan cell wall (Kadioglu et al., 2008). The chemical composition of the polysaccharide chains varies, resulting in at least 100 different *S. pneumoniae* serotypes. The capsule is a key virulence factor, in part due to its role in complement evasion; unencapsulated *S. pneumoniae* have low virulence and are rapidly removed from the circulation (Watson and Musher, 1990; Watson et al., 1995; Casal and Tarragó, 2003). The capsule blocks *S. pneumoniae* activation of the classical pathway by reducing bacterial recognition by natural and adaptive antibody, as well as direct binding of C1q and C reactive protein (Hyams et al., 2010). Furthermore the capsule inhibits activation of the alternative pathway and conversion of C3b to iC3b, and physically masks complement on the bacterial surface from binding to phagocyte complement receptors (Brown et al., 1983a; Joiner et al., 1983; Hyams et al., 2010). The result is that encapsulated *S. pneumoniae* are relatively resistant to complement-dependent phagocytosis (Hyams et al., 2010).

### Bacterial cell size and chain formation

A perhaps surprising but important concept about complement recognition of *S. pneumoniae* is the relative paucity of activation sites per bacterium. Instead of complement activation occurring evenly across the whole bacterial surface, there is focal formation of C3b/iC3b on the *S. pneumoniae* surface with a predilection for the site of divisional septa where the capsule layer is thinnest (Dalia and Weiser, 2011; Pathak et al., 2018). These foci of activated complement allow the spread of complement activation across the *S. pneumoniae* surface. As a consequence longer chains of *S. pneumoniae* are more susceptible to complement than short chains (Dalia and Weiser, 2011), providing one potential reason why *S. pneumoniae* is usually found as diplococci rather than in chains. Conversely, *S. pneumoniae* agglutination by antibody improves complement recognition as it allows the initial focal sites of complement activation to spread across multiple bacteria (Dalia and Weiser, 2011).

### Pneumolysin

The *S. pneumoniae* pore-forming toxin pneumolysin is released during bacterial growth and also found attached to the cell wall in some strains (Johnson, 1977; Balachandran et al., 2001; Price and Camilli, 2009). Extra-cellular pneumolysin activates the classical complement pathway by binding the F_c_ portion of IgG, diverting complement activation away from *S. pneumoniae* and increasing resistance to complement-mediated immunity (Paton et al., 1984; Mitchell et al., 1991; Yuste et al., 2005). In a mouse model the importance of pneumolysin for *S. pneumoniae* virulence was largely dependent on complement, and inhibition of both the alternative and classical complement pathways by a combination of pneumolysin and PspA respectively has a synergistic effect on *S. pneumoniae* complement evasion and virulence (Yuste et al., 2005).

### *S. pneumoniae* surface proteins

An increasing number of *S. pneumoniae* surface proteins have been described to inhibit complement activity against *S. pneumoniae* (Andre et al., 2017) (**Table 1**). The most notable are the cell wall choline binding proteins PspA and PspC which both have multiple roles for *S. pneumoniae* virulence, including complement evasion.

#### Choline binding proteins

PspA is expressed by all clinically important serotypes of *S. pneumoniae* and appears to inhibit both the classical and alternative complement pathways (Yuste et al., 2005), preventing complement-mediated clearance and phagocytosis of *S. pneumoniae* (Briles et al., 1997; Ren et al., 2004a; Quin et al., 2007; Ochs et al., 2008). In mouse infection models, PspA-dependent promotion of *S. pneumoniae* virulence is largely (but not completely) lost in mice deficient in C3, and both alternative and classical pathways contribute to this effect (Yuste et al., 2005). PspA is highly polar, with a strong electronegative charge on the region protruding through the cell wall that may directly impair complement deposition (Jedrzejas, 2001; Ren et al., 2004a). PspA also blocks classical pathway activity by preventing C-reactive protein binding to *S. pneumoniae* (Mukerji et al., 2012), and (in common with PspC), PspA can sequester the complement regulatory protein C4 binding protein (C4BP) from host serum (Haleem et al., 2019).

PspC binds factor H, a negative regulator of complement, thereby inhibiting factor B binding to C3b and accelerating the breakdown of the alternative pathway C3 convertase resulting in the inhibition of complement-dependent *S. pneumoniae* phagocytosis (Dave et al., 2004; Quin et al., 2005; Hammerschmidt et al., 2007; Li et al., 2007; Yuste et al., 2010). Assessing the relevance of PspC/factor H interactions in infection models has been impaired by the lack of PspC binding to rodent factor H (Lu et al., 2008). As discussed above, there is also evidence that PspC can also bind the classical pathway regulatory factor C4BP (Dieudonné-Vatran et al., 2009; Haleem et al., 2019).

Additional *S. pneumoniae* choline binding proteins may have a role in complement resistance, including murein hydrolases LytA and C, which are involved in remodelling of the cell wall and cell division. LytA binds both C4BP and factor H, inhibits C1q binding to CRP, and cleaves C3b and iC3b on the bacterial surface thereby inhibiting complement deposition by both the classical and alternative pathways (Ramos-Sevillano et al., 2015). LytC also seems to inhibit C3b binding to *S. pneumoniae* (Ramos-Sevillano et al., 2011).

#### Other surface proteins

There are several other *S. pneumoniae* surface proteins that might be involved in complement evasion (**Table 1**). These include the histidine triad proteins PhtA, PhtB, PhtD and PhtE, with deletion of all four Pht proteins increasing C3 deposition on *S. pneumoniae* serotype 4 although not other strains (Melin et al., 2010a). The proposed mechanisms include increased factor H binding (Ogunniyi et al., 2009; André et al., 2021) and/or direct degradation of C3 by PhtA (Angel et al., 1994; Hostetter, 1999; Zhang et al., 2001). Various *S. pneumoniae* biochemical enzymes affect complement activity including the exoglycosidases BgaA, Nan and StrH, which are thought to impair complement activity by deglycosylation of complement protein glycoconjugates (Dalia et al., 2010). Phosphoglycerate kinase can inhibit MAC formation (Blom et al., 2014), and the metallopeptidase endopeptidase O (PepO) cleaves plasminogen to plasmin which in turn cleaves C3b, thereby reducing C3b deposition on the *S. pneumoniae* surface (Agarwal et al., 2013; Agarwal et al., 2014). PepO has also been shown to interact with complement C1q, inhibiting the classical pathway (Agarwal et al., 2014).

## Variations in complement sensitivity between *S. pneumoniae* serotypes & strains

*In vitro* studies of complement activation of genetically engineered *S. pneumoniae* have demonstrated that capsular serotype causes very large differences in complement sensitivity (Hyams et al., 2010; Melin et al., 2010b). Opaque phase variants with a thicker capsule layer are more resistant to complement compared to transparent phase variants. However, capsular serotype effects on complement sensitivity are partially independent of capsule layer thickness (Hyams et al., 2013), implying the capsular carbohydrate structure is also important. This has been demonstrated for some specific serotypes: the related serotype 11A and 11E capsules only differ by O-acetylation of the galactose residue in the 11A capsule, but this results in ficolin-2 binding to the 11A capsule and activation of the lectin pathway whereas serotype 11E remains relatively complement resistant (Brady et al., 2014). Atomic force microscopy demonstrates that identical capsular serotypes expressed by *S. pneumoniae* and *Streptococcus mitis* have similar biomechanical properties, suggesting a relationship between capsule biochemistry and mechanical properties independent of bacterial strain. However, other factors must influence complement resistance as *S. mitis* strains were significantly more sensitive to complement than *S. pneumoniae* strains expressing the same capsular serotype (Marshall et al., 2020).

Along with capsular serotype, non-capsular variation in genetic background also determines differences between *S. pneumoniae* in their ability to resist complement (Hyams et al., 2011). Both *pspA* and *pspC* have marked allelic variation between *S. pneumoniae* strains (Croucher et al., 2017), and this is likely to cause at least some of the capsule-independent variation in complement sensitivity between strains. Indeed, the degree of factor H binding to *S. pneumoniae* dependent on PspC varies between strains (Yuste et al., 2010), with PspC subgroup 1 binding significantly more factor H than subgroup 2 (van der Maten et al., 2018). PspC subgroup 1 is the dominant subgroup in human cases of invasive *S. pneumoniae* infections (van der Maten et al., 2018) suggesting factor H binding to *S. pneumoniae* is potentially important for virulence. This is supported by data showing that PspC-dependent factor H binding variation between *S. pneumoniae* strains correlates with strain and capsular serotype ability to cause invasive infections in humans (Hyams et al., 2013). The association of complement-mediated phagocytosis with invasiveness within a pathogenic species suggests that complement resistance may be one important factor differentiating virulent from less virulent pathogens. This hypothesis is supported by data showing *S*. *mitis* strains were highly complement sensitive compared to *S. pneumoniae* (Marshall et al., 2020). However, increasing *S. mitis* resistance to complement by expression of a *S. pneumoniae* capsule or PspC (Rukke et al., 2014; Marshall et al., 2021) did not overcome the low virulence of *S. mitis* in mouse models of infection, indicating other factors are also important.

## Limitations of our understanding of *S. pneumoniae* interactions with complement

Characterization of *S. pneumoniae* protein interactions with complement have often relied on mutational analysis to demonstrate a role for a particular protein for complement evasion. However, care is necessary in interpreting these data - mutation of one gene can have direct and indirect effects on other genes or proteins, altering the complement resistance phenotype indirectly. Furthermore, the large degree of genetic variation between *S. pneumoniae* strains suggests the relative contributions of different complement evasion proteins will differ between *S. pneumoniae* strains. Lastly, even if a specific interaction between a *S. pneumoniae* protein and complement has been described it may not be functionally relevant; for example although other *S. pneumoniae* proteins have been shown to bind to factor H deletion of *pspC* results in complete loss of detectable factor H binding to multiple *S. pneumoniae* strains (Yuste et al., 2010; Hyams et al., 2013). There remain significant gaps in our understanding of the relative contributions of pneumococcal proteins for complement evasion during invasive disease in humans, partly reflecting the limitations in studying virulence factors using animal models, the complexity of the interplay between virulence factors, and the additional effects on pathogenesis of individual proteins beyond those related to complement.

## Conclusion

The complement system is central to the protective immune response to *S. pneumoniae*, with the balance between complement activation by host recognition of *S. pneumoniae* and complement evasion by multiple bacterial mechanisms significantly affecting the outcome of infection. Many questions remain about the interactions between *S. pneumoniae* and the complement system, including how capsule structure alters complement recognition, the exact roles during infection of the multiple non-capsular factors thought to inhibit complement activity, and the importance of complement for inflammatory and other non-phagocytic mechanisms of immunity to *S. pneumoniae*. A greater understanding of the mechanisms by which complement mediates immunity against *S. pneumoniae* and how *S. pneumoniae* evades these will provide a clearer understanding of disease pathogenesis, and more broadly potentially clarify why some bacterial commensal species are common pathogens and others rarely cause severe infections.

## Author contributions

EG, JB and MN conceived the manuscript. EG and JB wrote the text and figures, which was critically revised by MN. All have provided approval for publication.

## Funding

This work was supported by Wellcome Trust awards to EG (107311/Z/15/Z), MN (207511/Z/17/Z), and JB (221803/Z/20/Z). Meningitis Now and MRC awards (MR/S004394/1) to JB, and the National Institute for Health Research University College London Hospitals Biomedical Research Centre awards to MN and JB (IS-BRC-1215-20016 NIHR University College London Hospitals Biomedical Research Centre).

## Conflict of interest

The authors declare that the research was conducted in the absence of any commercial or financial relationships that could be construed as a potential conflict of interest.

## Publisher’s note

All claims expressed in this article are solely those of the authors and do not necessarily represent those of their affiliated organizations, or those of the publisher, the editors and the reviewers. Any product that may be evaluated in this article, or claim that may be made by its manufacturer, is not guaranteed or endorsed by the publisher.
